# The development and validation of the Japanese version of job satisfaction scale: a cross-sectional study on home healthcare nurses

**DOI:** 10.1186/s13104-022-06092-2

**Published:** 2022-06-15

**Authors:** Yoko Mori, Miki Sasaki, Yasuko Ogata, Taisuke Togari

**Affiliations:** 1 Department of Gerontological Nursing and Healthcare Systems Management, Graduate School of Health Care Sciences, Tokyo Medical and Dental University (TMDU), 1-5-45, Yushima, Bunkyo-ku, Tokyo, 113-8510 Japan; 2 Human Life and Health Sciences, Graduate School of Arts and Sciences, The Open University of Japan, Chiba-shi, Japan

**Keywords:** Home healthcare nurses, Job satisfaction, Japanese version, Scale development, Reliability, Validity

## Abstract

**Objective:**

A reliable and valid tool is required to assess home healthcare nurses’ job satisfaction for evaluating and improving the work environment and clinical practice of home healthcare. This study aimed to develop and examine the Japanese version of the Home Healthcare Nurses’ Job Satisfaction Scale (HHNJS-J). The Home Healthcare Nurses’ Job Satisfaction Scale (HHNJS) was translated into Japanese; a backward translation was performed until equivalence between the original and the backward-translated HHNJS was confirmed. Subsequently, a mail survey was conducted among 409 home healthcare nurses from 154 home healthcare agencies in Japan. We evaluated construct validity through Confirmatory Factor Analysis (CFA), and criterion-related validity and internal consistency were also tested.

**Results:**

The CFA revealed a second-order seven-factor structure and adequate internal consistency, although, the fit of the data to the factor structure was moderate. As per the goodness-of-fit indices of the final model of the CFA, the comparative fit index was 0.89 and root mean square error of approximation was 0.06. This newly translated scale can be used to assess the job satisfaction of home healthcare nurses within Japan. The HHNJS-J evaluated acceptable reliability and validity among Japanese home healthcare nurses and had application in clinical practice in Japan.

**Supplementary Information:**

The online version contains supplementary material available at 10.1186/s13104-022-06092-2.

## Introduction

With the aging population, the need for healthcare delivery is also increasing. Especially in developed countries, the mode of healthcare delivery is shifting from hospitals to community-based care [1, 2]. Therefore, medical care provided to older adults is becoming more sophisticated, and there is a greater need for high-quality home healthcare services to support community medical care [3].

In this context, to retain and increase human resources within the community, an attractive environment for nursing practice is necessary. Home healthcare nurses’ job satisfaction is highly associated with the quality and attractiveness of work environments and nurses’ intention to stay [4–6]. Over the last two decades, awareness regarding job satisfaction has been growing within international nursing research and practice [7]. Moreover, it has proven valuable for healthcare organizations as increased job satisfaction can improve nursing productivity and patient outcomes [8, 9]. Even in the domain of home healthcare nursing, job satisfaction enhanced by autonomy and control over work activities,improves patient outcomes and agency performance [10, 11]. However, the current literature on home healthcare nurses’ job satisfaction is limited [12–17]. Furthermore, most existing studies have been conducted among nurses working in acute care settings [6]. If the actual conditions of home healthcare agencies can be assessed by evaluating the job satisfaction of home healthcare nurses, it will help in the formulation of measures to retain them would be facilitated and nursing turnoverin community-based settings would be reduced.

Despite the limited existing literature, the development of the Home Healthcare Nurses’ Job Satisfaction Scale (HHNJS) [18] was initiated in 1998 to measure job satisfaction among home healthcare nurses. Modifications to improve internal consistency have been made by Ellenbecker, Byleckie, & Samia [18] who have further emphasized the need for reliable, valid, and useful tools. The final HHNJS was composed of 30 items across eight subscales (see Additional files 1, 2).

Our study aimed to confirm the reliability and validity of the Japanese version of the HHNJS (HHNJS-J).

## Main Text

### Methods

#### Design, participants, and survey methodology

This cross-sectional study included 409 Japanese home healthcare nurses who had been employed for more than 6 months by homecare agencies. The sample was recruited in two phases. First, we sent study cooperation requests to homecare agencies that were regular members of the National Association for Visiting Nurse Service in metropolitan areas including Tokyo. Then, we confirmed the number of copies of the questionnaire to be mailed to the agencies that agreed to cooperate with the study. Second, we sent the requested number of questionnaires to the homecare agency as well as an agreement for study cooperation. The selection was conducted from February to June 2020.

#### Development of the HHNJS-J

The original 30-item English scale was translated into Japanese. First, we gained permission to translate and use the HHNJS from the original author and the publisher [18]. Next, the principal researcher carried out an independent translation of the HHNJS from English to Japanese. Second, in the reconciliation phase, seven researchers reached a consensus on a draft of the Japanese translation of the HHNJS that best reflected the literal and conceptual equivalence with the English instrument. Third, in the backward translation phase, a professional translator, who is a native English speaker without knowledge of the original HHNJS, back-translated the Japanese version into English. Fourth, in the phase of back translation review and harmonization, the same seven researchers reviewed the back-translation to ensure it was conceptually equivalent to the original. The original author also confirmed the cognitive equivalence of the translated HHNJS-J.

#### Measurements

The questionnaire consisted of the HHNJS-J and an instrument to measure job satisfaction in Japanese nurses to evaluate criterion-related validity. We also collected information regarding the participants’ demographic characteristics.

##### HHNJS-J

The questionnaire included 30 items across eight subscales. Items were rated on a 5-point Likert scale ranging from 1 (*strongly disagree*) to 5 (*strongly agree*). Scores ranged from 30 to 150, with higher scores indicating greater job satisfaction. Five negatively worded items were reverse-scored.

##### Job satisfaction in Japanese Nurses Questionnaire

Nurses’ job satisfaction was also assessed using the 25-item Job Satisfaction Scale in Japanese Nurses Questionnaire [19, 20]where higher scores indicated greater satisfaction. Items were rated on a 5-point Likert scale ranging from 1 (*strongly disagree*) to 5 (*strongly agree*). Scores ranged from 25 to 125. This scale was reliable with a Cronbach’s alpha of 0.87 for the overall scale.

##### Demographic variables

These included age, gender, work status, educational background, period of working as a clinical nurse, period of working as a home healthcare nurse, family constitution of the person who required caregiving, and number of minor children.

#### Statistical analysis

Regarding participant characteristics, the proportion of categorical variables and mean,standard deviation, ceiling effect, and floor effect of each item were calculated [21].

Confirmatory factor analysis (CFA) was performed to test the fit of the data in relation to the factor structure. The original scale proposed a second-order eight-factor model which indicates the overarching concept of job satisfaction exists above the factors. This construct allows job satisfaction to be calculated using the total score of the scale, which is significant in creating the scale. Therefore,we performed CFA on the same construct as the original scale, but with several modifications to make the path diagram more faithful to the data extracted from Japanese home healthcare nurses. The modification resulted in four models: (1) a second-order eight-factor model: original HHNJS hypothesis model; (2) a second-order eight-factor model: model with changed affiliations; (3) a second-order seven-factor model; and (4) a second-order seven-factor model with an item deleted. Model fit was assessed using a combination of indices, including the goodness-of-fit index (GFI), comparative fit index (CFI), Tucker-Lewis index (TLI), and root mean square error of approximation (RMSEA). For the first two indicators, values > 0.90 were considered adequate [22–24], with a preference for values > 0.95 [25]. For the RMSEA index, values ≤ 0.05 indicated the best fit [26], although values between 0.05 and 0.08 indicated a reasonable fit [27, 28].

Pearson’s product-moment correlation coefficients were examined among the total scores in the Job Satisfaction in Japanese Nurses Questionnaire as an external criterion, the total score, and score for each subscale of a second-order seven-factor model with an item deleted to evaluate criterion-related validity.

Cronbach’s alpha was calculated for a second-order seven-factor model with an item deleted to assess internal consistency. Alpha coefficients ≥ 0.70 were considered satisfactory [29].

All statistical analyses, except for the CFA, were performed using SPSS 26 for Windows (IBM Corp., Armonk, NY, USA). CFA was conducted using AMOS version 26 for Windows (Chicago, IL; IBM SPSS Statistical Programs). Statistical significance was set at *p* < 0.05.

## Results

### Sample characteristics

A total of 154 homecare agencies responded to the study cooperation requests (response rate = 7.7%) and 446 respondents returned the questionnaire (response rate = 53.4%). Of these, 37 respondents were excluded because of incomplete answers to the HHNJS-J or Job Satisfaction in Japanese Nurses Questionnaire. The remaining 409 responses were included in the analysis. The sociodemographic characteristics of the respondents are shown in Table 1. The mean total HHNJS-J score was 93.4 (standard deviation = 12.1, range 26–130). None of the items demonstrated a ceiling or floor effect (Table 2).

**Table 1:** Participants' sociodemographic characteristics (n = 409)

Variables	n/(Mean)	%/[SD]
Gender		
Women	393	96.1
Men	15	3.7
Missing	1	0.2
Age (Years)		
< 30	23	5.6
< 40	70	17.1
< 50	150	36.7
< 60	133	32.5
≧60	32	7.8
Missing	1	0.2
Period of working as a clinical nurse (Years)		
< 1	1	0.2
< 5	33	8.1
< 10	70	17.1
< 20	134	32.8
≧20	169	41.3
Missing	2	0.5
Educational background		
Vocational school	284	69.5
Junior college	37	9.0
Bachelor's degree or higher	63	15.4
Others	24	5.9
Missing	1	0.2
Period of working as a home healthcare nurse (Years)		
< 1	30	7.3
< 5	154	37.6
< 10	92	22.5
< 20	132	32.3
Missing	1	0.2
Work status		
Full-time	294	71.9
Part-time	114	27.9
Missing	1	0.2
Family constitution		
Alone	53	13.0
Living with families requiring caregiving or having underage children	192	46.9
Living separately from families requiring caregiving or having underage children	163	39.9
Missing	1	0.2
HHNJS-J: Model 4	(93.4)	[12.1]
Job Satisfaction in Japanese Nurses Questionnaire	(89.2)	[11.5]

HHNJS-J: Japanese version of the Home Healthcare Nurses' Job Satisfaction Scale

**Table 2:** Means, standard deviations, ceiling effects, floor effects, and Cronbach's Alphas for the total Scale and Model 4 factors (n = 409)

Items of HHNJS-J Model 4 (α = .91) and factors with Cronbach’s alphas		Mean	SD	Ceiling effect	Floor effect
Relationship with patients (α = 0.81)					
1	Patients are satisfied with the care that I provide	3.64	0.59	4.23	3.05
2	The relationships I have built with patients are valuable	3.99	0.51	4.49	3.48
3	I am helping to maintain or improve patients’ quality of life	3.97	0.45	4.42	3.52
4	My job is important and fulfilling	4.08	0.70	4.77	3.38
5	The patient care that I provide conforms to professional standards (ethical norms and accountability)	3.97	0.54	4.51	3.43
24	I can carry out every task that my job requires	3.57	0.73	4.31	2.84
Relationship with colleagues (α = 0.90)					
6	Being able to get support from my colleagues is a good aspect of my job	4.13	0.62	4.74	3.51
7	I am getting along with the nurses with whom I work	4.14	0.68	4.81	3.46
8	I have a cooperative relationship with the nurses with whom I work	4.09	0.72	4.80	3.37
9	I have colleagues whom I can trust and rely on	4.17	0.72	4.89	3.46
Professional pride (α = 0.86)					
10	If I were to choose my specialty again, I would probably choose home health nursing	3.66	1.04	4.70	2.62
11	I would like to recommend my job to other health professionals	3.69	0.94	4.63	2.75
12	I speak with pride when I discuss my job with others	3.95	0.86	4.80	3.09
Relationship with doctors (α = 0.81)					
13	Doctors respect my opinions regarding home care patients	3.42	0.79	4.20	2.63
14	Doctors treat me as a colleague, a nursing specialist	3.26	0.85	4.12	2.41
Relationship with institution (α = 0.76)					
15	I am content with the professional relationship that we have with the nurse administrator of this facility	3.56	0.90	4.46	2.65
16	I have some influence on organizational policy changes at this facility	2.92	1.05	3.97	1.87
17	I have the opportunity to develop skills that will advance my nursing expertise at this facility	3.49	0.89	4.38	2.60
25	I can handle the growing demand for documentation in home health nursing	3.22	0.80	4.02	2.43
Autonomy and control (α = 0.78)					
18	I can adjust my working hours if necessary	3.65	0.93	4.58	2.72
19	I can change my working hours more flexibly than other clinical nurses	3.53	0.99	4.52	2.54
20	I can adequately manage my time outside of work	3.45	0.93	4.39	2.52
21	I independently make important decisions in my daily work	3.64	0.84	4.47	2.80
Salary and benefits (α = 0.62)					
27	I am satisfied with my current salary	2.84	1.09	3.92	1.75
28	The pay scale at this facility needs improvement	2.52	0.96	3.47	1.56
30	I am satisfied with the employee benefits provided at this facility	2.85	1.02	3.87	1.84

*HHNJS-J* Japanese version of the Home Healthcare Nurses' Job Satisfaction Scale, *SD* standard deviation

### Structure of the HHNJS-J

The following four-factor analysis models were specified and compared (see Additional file 3, Table S1).

#### Model 1: Second-order eight-factor model: original HHNJS hypothesis model

First, a second-order eight-factor model was tested. This model included all 30 items of the original HHNJS hypothesis model. However, this model showed a relatively poor fit with the data.

#### Model 2: Second-order eight-factor model: a model with changed affiliations

Next, a second-order eight-factor model was tested, according to the results of Model 1. We changed the affiliation factors attributed to items 24 and 25 according to the factor loadings of the exploratory factor analysis.

#### Model 3: Second-order seven-factor model

Next, a second-order seven-factor model was tested, according to the results of Model 2. Path coefficients between first-order; “job satisfaction” and second-order; “stress and workload” were 0.00, which implies no association at all. Hence, we removed “stress and workload” from the factors, which means that it generated a seven-factor structure. This modification resulted in a different structure of the original eight-factor model.

#### Model 4: Second-order seven-factor model: model with one item deleted

Notably, the second-order seven-factor model served as a boundary model for the viability of the more elaborate model. One concern was the path coefficient of item 29 (stress and workload), which was -0.06. Therefore, this item was deleted.

After releasing two error term covariances based on the largest and second largest modification indices, the model fit indices were CFI = 0.885, GFI = 0.853, TLI = 0.873, and RMSEA = 0.064 [95% CI 0.060–0.069].

**Fig. 1:**
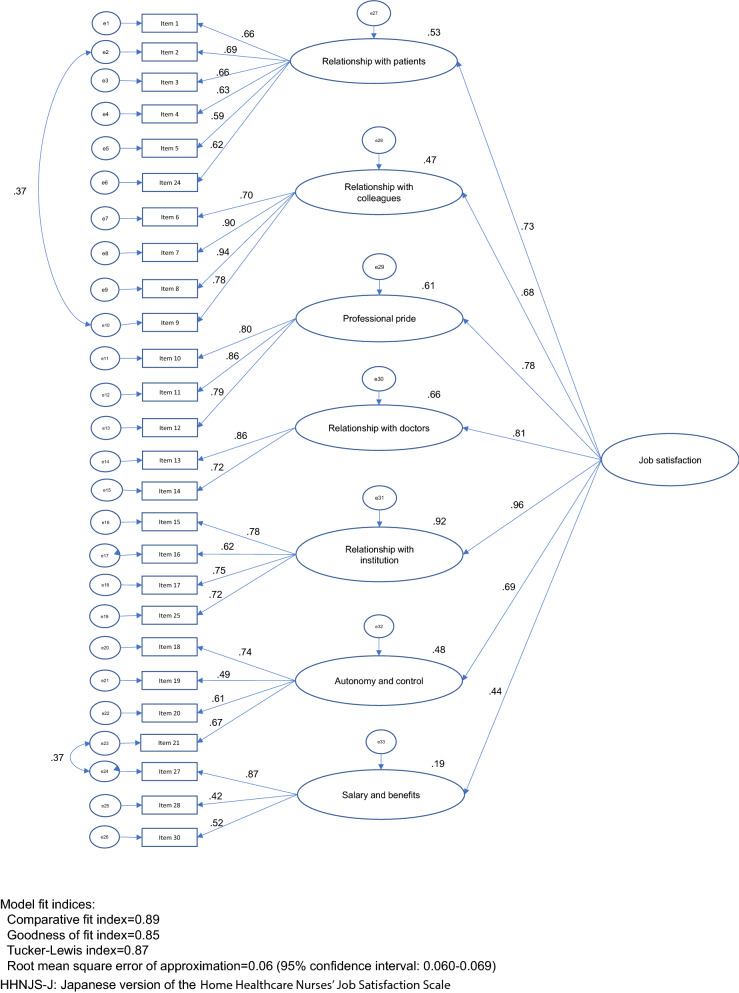
Confirmatory factor analysis of the HHNJS-J (Model 4). Model fit indices: Comparative fit index = 0.89, Goodness-of-fit index = 0.85, Tucker-Lewis index = 0.87, Root mean square error of approximation = 0.06 (95% confidence interval 0.060–0.069). *HHNJS-J* Japanese version of the Home Healthcare Nurses’ Job Satisfaction Scale

While we remained faithful to the structure of the original scale, we made modifications based on the results of our data. Thus, Model 4 was proposed (Fig. 1).

### Criterion-related validity of the HHNJS-J: Model 4

The correlations among the total score of the HHNJS-J: Model 4 and the scores of the individual factor; relationship with patients, colleagues, doctors, and institution; professional pride; autonomy and control; and salary and benefits were significantly positively correlated with the scores on Job Satisfaction in Japanese Nurses Questionnaire (see Additional file 4, Table S2).

### Reliability of the HHNJS-J: Model 4

The Cronbach’s alpha coefficients of the total HHNJS-J and each factor is shown in Table 2. Cronbach’s alpha of the total HHNJS-J was 0.91. Cronbach’s alpha coefficients of the seven factors were: 0.81 (relationship with patients), 0.90 (relationship with colleagues), 0.86 (professional pride), 0.81 (relationship with doctors), 0.76 (relationship with institution), 0.78 (autonomy and control), and 0.62 (salary and benefits).

## Discussion

The results of the CFA to test the fit of the data to the factor structure revealed moderate structural validity. Among the four models, Model 4 was a better fit than Models 1–3. The difference between the original scale and the HHNJS-J in terms of factor structure is expected to be based on cross-cultural and other differences among the participants. In this study,the item “salary at other facilities is higher than at this one,” included in factor seven of “salary and benefits,” was consequently deleted. Possible causes include; (1)home healthcare nurses in Japan might not know salary at other facilities; (2) they might not be interested in salary, because communicating with clients was highly valued [30]. In contrast, the satisfaction of home healthcare nurses outside of Japan has been linked to stable and high income, with greater satisfaction associated with salary and benefits, which has been reported to have a positive correlation with their intent to remain employed [31].

The item “Sometimes, I experience stress from the fact that my duties are predetermined,” included in the factor of “stress and workload,” was consequently deleted. In the US, autonomy and independence have been shown to be important for homecare nurses’ job satisfaction [32]. In contrast, home healthcare nurses in Japan do not require a high degree of independence. In a previous study, the factors that most influenced job satisfaction among home healthcare nurses in Japan were approval from clients and clients’ families, and relationships within the workplace [33].

Although this scale was a faithful replication of the original, the results indicate the need to modify some items from a cross-cultural perspective. Thus, further studies are needed to investigate the construct of job satisfaction among home healthcare nurses in Japan. Overall,the results of this study encourage and promote the use of this translated scale in home healthcare settings in Japan. These findings would contribute towards policy change directed at improving job satisfaction among home healthcare nurses.

## Limitations

There are two main limitations to this study. First, generalizability is limited owing to the sampling method. Samples were collected only in metropolitan areas including Tokyo. Therefore, caution must be exercised in attempting to generalize the findings to different Japanese populations. Second, the degree of fitness of the CFA was insufficient. The cross-cultural differences between the original HHNJS and HHNJS-J may require elaboration of scale items and further research.

## Supplementary Information

**Additional file 1:** The Home Healthcare Nurses' Job Satisfaction Scale: HHNJS.

**Additional file 2:** Item analysis and exploratory factor analysis of 30 items (same as original HHNJS Scale) Japanese version exploration.

**Additional file 3: Table S1.** Results of confirmatory factor analysis: comparison of several indices from Models 1 to 4 (n = 409)

**Additional file 4: Table S2.** Pearson’s correlation coefficients between the satisfaction in Japanese Nurses' questionnaire and the total and factor Scores of the HHNJS-J: Model 4 (n = 409)

## Data Availability

The datasets generated during and/or analyzed during the current study are available from the corresponding author on reasonable request.

## Abbreviations

HHNJS: The Home Healthcare Nurses’ Job Satisfaction Scale
HHNJS-J: Japanese version of the Home Healthcare Nurses’ Job Satisfaction Scale
CFA: Confirmatory factor analysis
GFI: The goodness-of-fit index
CFI: Comparative fit index
TLI: Tucker-Lewis index
RMSEA: Root mean square error of approximation
95% CI: 95% Confidence interval
χ2/df: Chi- square to degrees of freedom
